# Germline Variation Controls the Architecture of Somatic Alterations in Tumors

**DOI:** 10.1371/journal.pgen.1001136

**Published:** 2010-09-23

**Authors:** Amy M. Dworkin, Katie Ridd, Dianne Bautista, Dawn C. Allain, O. Hans Iwenofu, Ritu Roy, Boris C. Bastian, Amanda Ewart Toland

**Affiliations:** 1 Integrated Biomedical Sciences Graduate Program, The Ohio State University, Columbus, Ohio, United States of America; 2 Department of Dermatology and UCSF Helen Diller Family Comprehensive Cancer Center, University of California San Francisco, San Francisco, California, United States of America; 3 Singapore Clinical Research Institute, Singapore; 4 Clinical Cancer Genetics Program and Human Cancer Genetics Program, Department of Internal Medicine, Division of Human Genetics, Arthur G. James Cancer Hospital and Richard J. Solove Research Institute, The Ohio State University Medical Center, Columbus, Ohio, United States of America; 5 Department of Pathology and Laboratory Medicine, The Ohio State University Medical Center, Columbus, Ohio, United States of America; 6 Biostatistics Core Facility, UCSF Helen Diller Family Comprehensive Cancer Center, University of California San Francisco, San Francisco, California, United States of America; 7 Departments of Dermatology and Pathology and UCSF Helen Diller Family Comprehensive Cancer Center, University of California San Francisco, San Francisco, California, United States of America; 8 Departments of Internal Medicine and Molecular Virology, Immunology, and Medical Genetics, Divison of Human Cancer Genetics, Comprehensive Cancer Center, The Ohio State University, Columbus, Ohio, United States of America; University of Washington, United States of America

## Abstract

Studies have suggested that somatic events in tumors can depend on an individual's constitutional genotype. We used squamous cell carcinomas (SCC) of the skin, which arise in high multiplicity in organ transplant recipients, as a model to compare the pattern of somatic alterations within and across individuals. Specifically, we performed array comparative genomic hybridization on 104 tumors from 25 unrelated individuals who each had three or more independently arisen SCCs and compared the profiles occurring within patients to profiles of tumors across a larger set of 135 patients. In general, chromosomal aberrations in SCCs were more similar within than across individuals (two-sided exact-test p-value 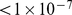), consistent with the notion that the genetic background was affecting the pattern of somatic changes. To further test this possibility, we performed allele-specific imbalance studies using microsatellite markers mapping to 14 frequently aberrant regions of multiple independent tumors from 65 patients. We identified nine loci which show evidence of preferential allelic imbalance. One of these loci, 8q24, corresponded to a region in which multiple single nucleotide polymorphisms have been associated with increased cancer risk in genome-wide association studies (GWAS). We tested three implicated variants and identified one, *rs13281615*, with evidence of allele-specific imbalance (p-value = 0.012). The finding of an independently identified cancer susceptibility allele with allele-specific imbalance in a genomic region affected by recurrent DNA copy number changes suggest that it may also harbor risk alleles for SCC. Together these data provide strong evidence that the genetic background is a key driver of somatic events in cancer, opening an opportunity to expand this approach to identify cancer risk alleles.

## Author Summary

Tumors exhibit DNA copy number gains and losses, many of which alter the dosage of genes that promote or suppress tumorigenesis. Evidence from familial cancer syndromes and animal models have shown that DNA copy number changes acquired somatically during tumor progression can be controlled by the constitutional genotype. The genetic heterogeneity among humans makes it difficult to systematically assess the extent of this effect. We used a unique clinical scenario of squamous cell carcinoma (SCC), which can arise in high multiplicity within patients, to compare the pattern of somatic alterations on a homogeneous genetic background. We examined the genome-wide pattern of DNA copy number changes of tumors from individuals who had three or more independent SCCs. We identified multiple chromosomal regions that showed higher frequency of change in SCCs within patients than across patients, suggesting that the genetic background of the individual is important in driving these changes. We further confirmed this by identifying eight regions with strong evidence for a selection of loss or gain of a particular allele within patients. Together these data demonstrate that the genetic background of an individual influences the pattern of somatic alterations in tumors, offering a novel approach to map susceptibility alleles.

## Introduction

Human solid cancers are characterized by the presence of numerous genetic alterations that accumulate during the evolution of the disease. While the mutation spectrum within biologically related cancer subtypes often shows similarities with regards to the patterns of genetic alteration, each individual cancer has a unique combination of alterations. The forces that shape the genomic landscape of individual cancers are in part determined by the nature of the initiating oncogenic alterations and the sequence in which they occur. However, the constitutional genotype of the cell acquiring the first pathogenetically relevant mutation is likely to play a role in influencing which somatic alterations will undergo positive or negative selection. The influence of inherited alterations on the pattern of somatic mutations found in evolved cancers has been demonstrated in several cancer types. In breast cancers from individuals with inherited *BRCA1* mutations one finds more frequent losses on 4p, 4q, 5q, Xp and Xq and gains of 10p and 16q compared to breast tumors from individuals without *BRCA1* mutations [1], [2]. In melanoma, patients with germline variations in *MC1R* have a higher frequency of somatic *BRAF* mutations in their melanomas than patients without *MC1R* variants [3], [4]. These examples of interactions between predisposing germline alterations and acquired mutations in the tumor occur between different genes (trans-effects). Several studies have also identified cis-effects, in which somatic alterations affect specific inherited variants. Examples include two genes identified through mouse mapping studies: *AURKA*, which shows allele-specific gains of the T91A allele in human colon tumors [5], [6] and *PTPRJ*, which shows allele-specific losses of the A1176C allele in human colon tumors [7]. In addition, *rs6983267*, a SNP on 8q24 found through several genome-wide association studies to be associated with susceptibility to colorectal cancer, shows allele-specific imbalance [8].

Together these data suggest that inherited variation as well as somatic mutations arising early in progression help shape the pattern of somatic changes that occur subsequently during tumor evolution. One way to more systematically assess the effect of the constitutional genotype on the pattern of somatic alterations is to compare cancers of the same type that arose independently on the common genetic background of a single individual. Only a few cancer types arise frequently enough to render such analysis practical. Basal cell carcinoma and squamous cell carcinoma (SCC) of the skin often develop in multiplicity. Furthermore, the incidence of SCC in particular is dramatically increased in immunosuppressed patients. Specifically, in organ transplant recipients (OTRs) the risk of SCC is 65 to 250 fold increased compared to the general population [9]. As a consequence, some patients develop dozens of separate primary carcinomas. In this study, we exploited the unique property of the OTR population to test the hypothesis that tumors arising on a common genetic background will have somatic alterations that are more similar to each other than to those found in similar tumors that developed in different individuals and whether this scenario can be exploited to discover predisposing genetic factors.

## Results

**Figure 1:**
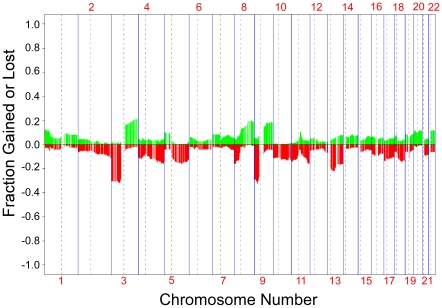
Frequency of aCGH aberrations in skin tumors. A frequency plot of somatic aberrations identified in 305 skin tumors by aCGH is shown. Each line is an individual BAC clone. Green indicates gain and red indicates loss. Clones are ordered from chromosome 1 through 22. Tumors profiled are from 181 patients with one or more independent SCCs and/or KAs.

We obtained copy number profiles as measured by array comparative genomic hybridization (aCGH) from tumors arising in individuals with multiple independent cutaneous squamous cell carcinomas (SCCs) or keratocanthomas (KAs) (intra-group) and copy number profiles of SCCs and KAs of separate individuals (inter-group). 305 independent tumor samples from 181 patients were included in this initial study (Figure 1). As previously reported [10], [11], focal genomic aberrations were rare in these tumors and DNA copy number aberrations consisted mostly of the loss or gain of whole chromosome arms. As the resolution of copy number changes using aCGH is around 1Mb, it is possible that we missed focal amplifications or deletions in this study.

We compared aCGH profiles between the three types of skin tumors in our study, SCC, SCC *in situ* (Bowen's Disease) and keratocanthoma. There were no statistically significant differences in frequency of clone loss or gain between the SCC and keratocanthoma profiles; however there were several loci which showed differences between the SCC *in situ* profiles and profiles from the other two tumor types (data not shown). Because of this, we focused our comparative analysis on SCCs only. Our data set included 222 SCCs from 135 individuals. From 25 of those individuals, three or more SCCs (median = 4.2; range 3–6) were analyzed to compare the intra-group and inter-group similarities of DNA copy number changes.

**Figure 2:**
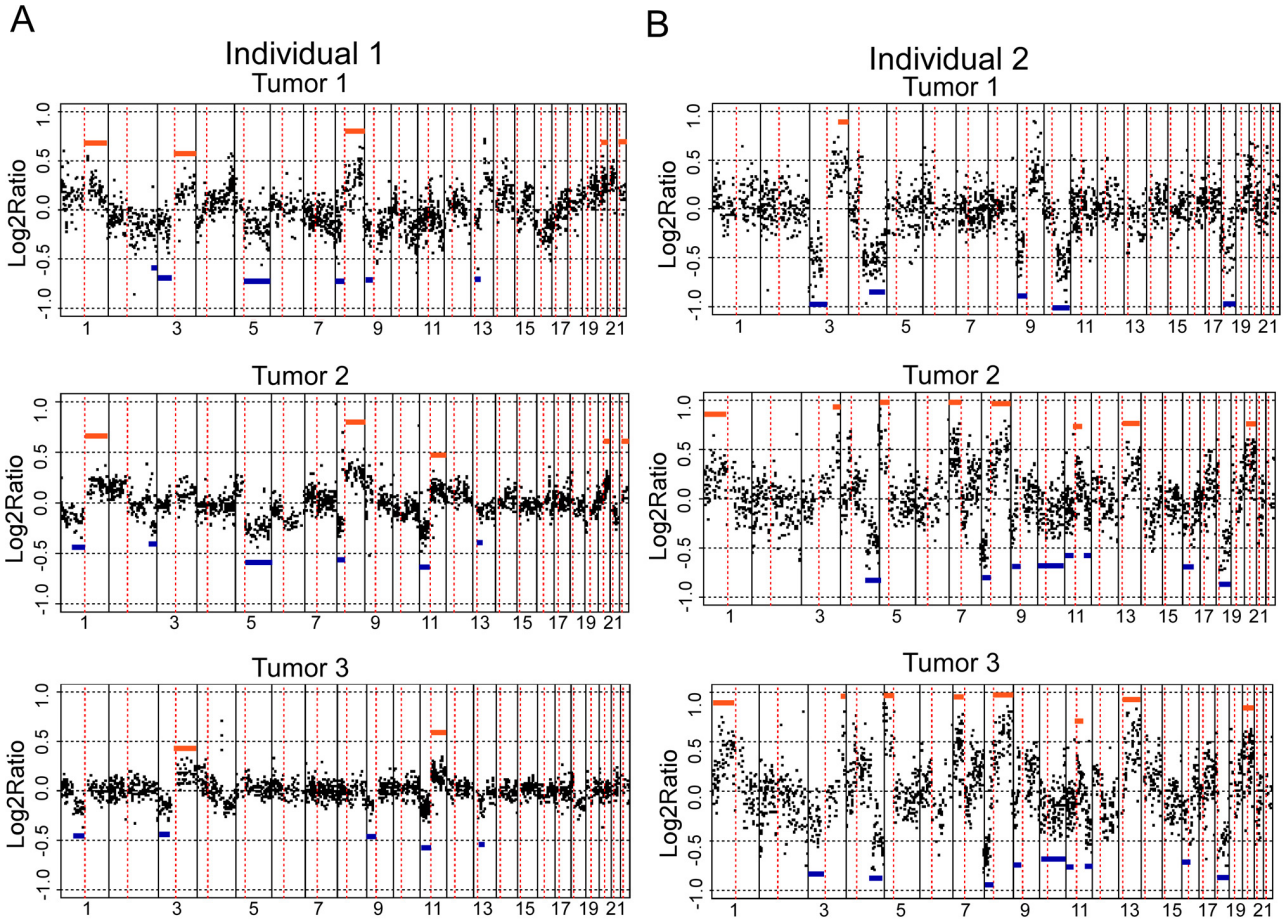
aCGH profiles of independent tumors from two individuals. aCGH profiles of three independent tumors from two individuals, A and B, are shown. Each dot represents a different BAC clone. The X-axis for each profile shows the BAC clones ordered from chromosomes 1 through 22. Chromosome boundaries are indicated by vertical lines and dotted lines indicate centromeres. The Y-axis is the log2ratio of the tumor genomic DNA compared to reference DNA. Blue lines indicate regions showing concordance for loss and orange lines indicate regions showing concordance for gain across tumors.

We found a significantly higher concordance of chromosomal aberrations in SCCs within than between patients [two-sided T-test p-values: 6.97×10^−8^](Figure 2). Interestingly, certain chromosomal regions (4q, 11q, and 17q) were preferentially affected by this concordance (individual arm p-values<0.05; Table 1). The intra-group correlation coefficients (ICC) for the array elements of these regions were compared but did not allow narrowing the genomic region to specific loci within these regions. This is not unexpected, considering that most of the tumors showed copy number changes affecting large genomic regions, such as entire chromosomal arms or chromosomes.

**Table 1:** Comparison of somatic changes within versus across individuals.

Region	Frequency Gains	Frequency Losses	Mean Correlation Coefficient Value Within Patients	Mean Correlation Coefficient Value Between Patients	P-value*
1p	6%	3%	0.25	0.16	0.2
1q	8%	2%	0.25	0.08	0.22
2q	1%	6%	0.30	0.14	0.22
3p	1%	32%	0.36	0.27	0.31
4q	3%	12%	0.29	0.1	0.003
5q	1%	14%	0.03	0.15	0.30
8p	4%	15%	0.27	0.18	0.31
8q	12%	1%	0.33	0.20	0.25
10q	1%	9%	0.15	0.04	0.27
11q	5%	9%	0.41	0.23	0.02
13q	7%	15%	0.15	0.04	0.30
14q	6%	2%	0.14	0.18	0.93
17q	4%	9%	0.30	0.09	0.008
20q	7%	1%	0.23	0.19	0.93

*Holm's adjusted t-test for unequal variance (with Welch's approximation) p-value.

**Figure 3:**
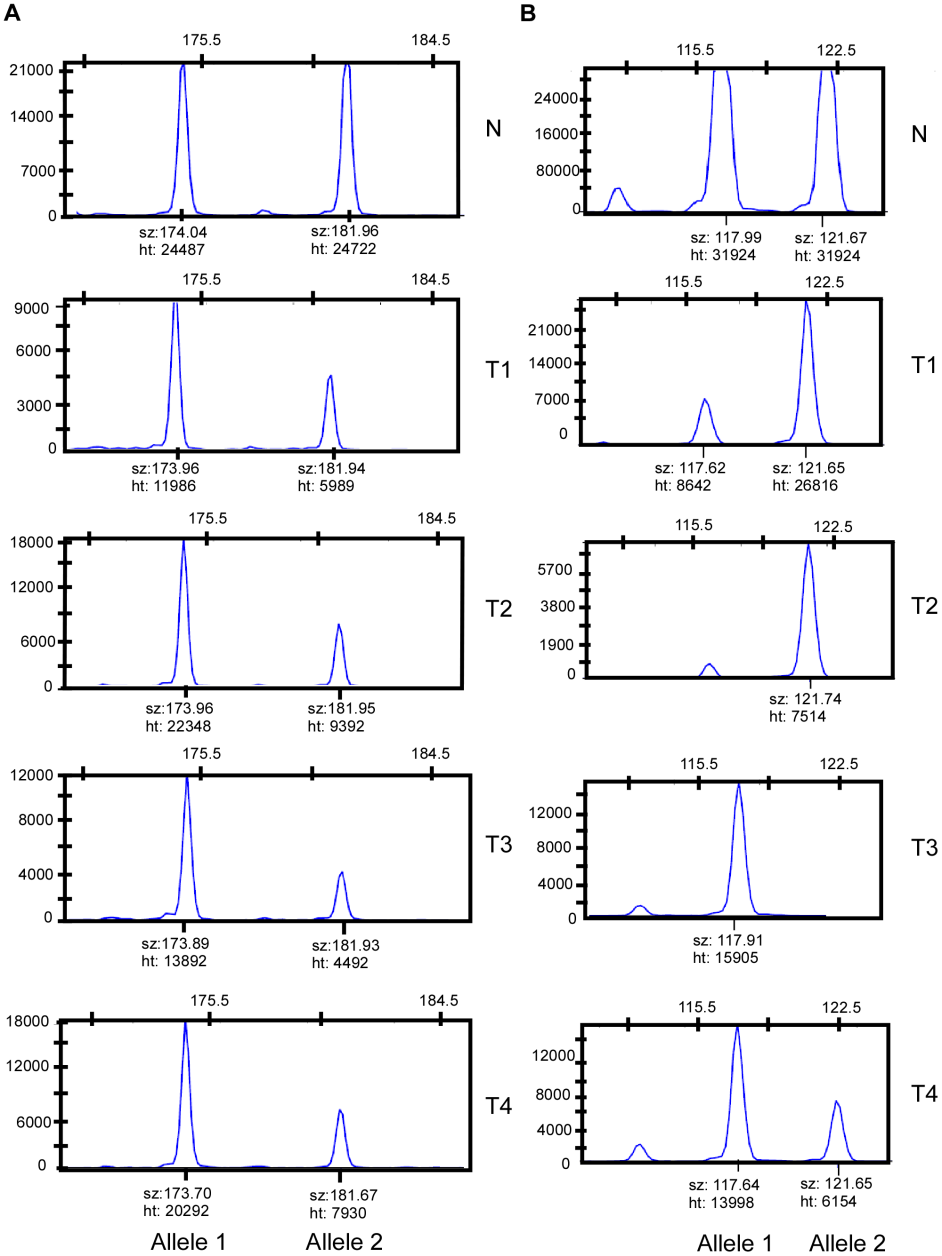
Loci with and without evidence of preferential allelic imbalance. (A) Microsatellite marker showing evidence of preferential allelic imbalance. Four tumors from one individual were typed for microsatellite marker D3S3045. Compared to blood DNA, all four tumors showed respective loss of the 182 bp allele. A pattern of preferential allelic imbalance was observed in 8 of 9 informative individuals for this marker. (B) Microsatellite marker showing random allelic imbalance. Four tumors from one individual were typed for microsatellite marker D11S4463. Compared to the matched blood DNA, two tumors showed relative loss of the 118 bp allele and two tumors showed relative loss of the 122 bp allele.

We rationalized that any inherited variants that promote cancer in an allele-specific manner would result in allele-specific DNA copy number changes reflected by preferential loss or gain of one specific chromosome in the tumors of an individual patient. By contrast, dosage events affecting genes that promote cancer in allele-independent manner, e.g. loss of *CDKN2A* or gain of *MYC*, were expected to display random somatic alterations of either allele [12]. To determine the presence of allele-specific changes occurring within tumors of individual patients, we performed loss of heterozygosity analyses of 45 microsatellite markers covering 14 chromosomal regions that were chosen based on the frequency of aberration as measured by aCGH and without prior knowledge of regions showing more similarity within versus across patients. 270 tumors from 65 individuals with a minimum of three independent tumors were included in this analysis. The constitutional genotype was determined from DNA extracted from blood leukocytes of each patient. Allelic imbalance was defined as a tumor to normal DNA allelic ratio of greater than 1.5 or less than 0.67. Statistical analyses for preferential imbalance were conducted for individuals who were heterozygous for a given marker and had two or more tumors showing imbalance; two examples are illustrated in Figure 3. Thirteen markers representing eight different genomic regions showed significant skewing towards one allele as determined by a Bayesian/frequentist approach (Text S1). Markers demonstrating significant preferential allelic imbalance mapped to chromosomal locations 3p24, 3q21-26, 5q23, 7p12-21, 7q31, 8q24, 9p21, 11q24, and 18q22 (Table 2, Figure 3). These data indicate that the increased similarity of copy number changes within individuals is at least in part due to inherited variation within the same region as the copy number change.

**Table 2:** Preferential allelic imbalance studies.

Locus	Marker	Number of patients			p-value
		Heterozygous	Multiple tumors with LOH (tumor number)	Preferential imbalance*	
1p35.1-1p34.3	D1S3720	27	5 (12)	2	0.63
1p35.1-p34.3	D1S3721	28	4 (11)	1	0.25
3p26.1	D3S4545	35	12 (39)	11	0.008
3p24	D3S3038	34	15 (58)	11	0.007
3p21.2-p14	D3S1766	37	16 (73)	12	0.007
3p21.2-p14	D3S3644	43	14 (49)	10	0.10
3p21.2-p14	D3S4529	10	7 (18)	6	0.03
3q21-3q29	D3S3045	51	9 (34)	8	0.02
3q21-q29	D3S1746	44	19 (62)	11	0.37
3q21-q29	D3S1311	41	14 (49)	9	0.45
5q23	D5S2501	21	14 (41)	11	0.05
5q23	D5S1505	26	17 (51)	5	0.10
5q23	D5S816	25	18 (66)	9	0.73
7p21.1	D7S638	19	4 (11)	1	0.25
7p21.1-p15.3	D7S503	27	7 (24)	6	0.05
7p12	D7S1818	26	10 (39)	9	0.04
7p21.11-p21.12	D7S644	12	1 (12)	1	1.00
7q22.1	D7S1799	13	1 (3)	1	1.00
7q22.3	D7S2420	16	4 (9)	4	0.13
7q31.1	D7S2418	25	7 (29)	7	0.02
7q31.2	D7486	24	7 (23)	4	0.47
7q31.33	D7S1873	25	15 (38)	9	0.39
8p12	D8S1048	22	10 (38)	5	0.13
8p12	D8S1477	33	15 (45)	11	0.15
8q22-q24	D8S1132	45	8 (32)	7	0.02
8q24.1	D8S1128	40	11 (31)	6	0.82
8q24.3	D8S373	39	11 (26)	7	0.94
9p21	D9S925	44	25 (71)	20	0.02
9p21	D9S1118	35	10 (30)	5	1.00
9q	D9S934	30	10 (28)	8	0.12
9q	D9S1825	32	8 (23)	5	0.48
9q	D9S2157	8	2 (4)	2	0.5
9q	D9S1838	34	17 (44)	6	0.54
11q23-q25	D11S1986	39	20 (50)	14	0.03
11q23-q25	D11S4463	27	12 (16)	9	0.17
11q23-q25	D11S969	22	13 (23)	9	0.18
13q12-q21	D13S1493	31	20 (72)	13	0.85
13q14.1	D13S155	21	12 (36)	5	0.34
13q12-q21	D13S800	20	14 (42)	8	0.85
13q12-q21	D13S796	32	15 (45)	8	0.18
13q12-q21	D13S285	32	25 (66)	12	0.80
17p13.1	D17S974	18	11 (28)	9	0.12
18q22-q23	D18S1364	24	14 (44)	5	0.52
18q22-q23	ATA82B02	24	18 (64)	12	0.01
18q22-q23	D18S1390	16	8 (27)	2	0.67

*ln O_j_<−1.5.

The next question we addressed was whether variations in any known tumor susceptibility genes were driving allele-specific imbalance at the loci identified through our studies. Several genome-wide association studies (GWAS) have been performed for multiple cancers including breast, prostate, colon, and melanoma [13]–[15]. Variants at 8q24 identified via GWAS have been associated for cancer risk for multiple cancer types [8], [13], [16]–[21]. To determine if any of these were candidates for the observed allele-specific imbalances at 8q24, we tested three variants, *rs13281615*, *rs1447295*, and *rs6983267*, for allele-specific imbalances in matched normal and tumor DNAs from individuals with SCC. Of these, only *rs13281615* showed statistically significant evidence of allelic skewing (Table 3). Of 35 heterozygous tumors showing imbalance for *rs13281615*, 28 of them showed an imbalance in favor of the A allele while only 7 showed an imbalance favoring the G allele (p-value 0.012). A second SNP, *rs6983267*, showed a similar trend that did not reach statistical significance (p-value 0.157). These data raise the possibility that *rs13291615* may be a candidate susceptibility allele for SCC. Our results suggest that the use of preferential allelic imbalance may be an efficient approach to map susceptibility variants in specific clinical settings.

**Table 3:** 8q24 variants tested for preferential allelic imbalance.

SNP	Location on 8q24	Heterozygous tumors showing imbalance	“Retained”allele in SCCs	“Lost” allele in tumors	Chi-square p-value	GWAS Study
*rs13281615*	128355618	35	28	7	0.012	Breast [13]
*rs6983267*	128413305	16	12	4	0.157	Colon; prostate [21], [34]
*rs1447295*	1284845038	6	3	3	1	Prostate [21]

SNP, single nucleotide polymorphism; GWAS, genome-wide association study.

## Discussion

In summary, our finding of an increased concordance of DNA copy number changes together with the presence of allelic-specific imbalance within separate cancers of individuals strongly suggests that the somatic changes occurring in tumors are in part affected by underlying characteristics of the individual host. An in depth comparison of allele-specific genomic changes occurring in multiple tumors of individual patients may offer a unique route to uncover cancer susceptibility loci.

The allele-specific LOH data from both microsatellite analysis and from SNP analysis indicate that the increased similarity of copy number changes within individuals is at least in part due to inherited variation within the same region as the copy number change. By contrast, not all loci that were frequently affected by concordant aberrations within individuals showed evidence of preferential allelic imbalance. This could be due to trans-effects between inherited variants elsewhere in the genome and a cancer gene in the region affected by the copy number alteration. For example, 13q12-q21, containing the *Rb* tumor suppressor gene, showed frequent loss in SCCs and a high intra-group concordance but did not show evidence of preferential imbalance.

There are some alternative explanations for the greater similarity of changes in tumors within versus between individuals. In our study we defined tumors as being independent based on arising in different anatomical sites. This should reduce the probability that tumors are related via a shared clonal origin. It is unlikely, but not impossible that tumors arising on different sites might have a common precursor which would explain the results of this study. It also remains possible that other pathogenetic factors such as ultraviolet light exposure or the presence of human papilloma virus may also influence the similarity of somatic alterations of tumors arising within an individual that do not show allele-specific imbalance. Finally, different immunosuppressive drugs may result in specific mutations occurring in tumors which might manifest as similar copy number patterns in tumors from within an individual. Another explanation of our results is that environmental exposures may result in differential selection between alleles which could result in allele-specific imbalances. Despite these possibilities, our study strongly supports the notion that the constitutional genotype of an individual exerts a strong influence on the somatic alterations that arise in cancer. Genetic analyses of cancer that arise at high multiplicity may offer a novel route to the discovery of cancer susceptibility genes.

## Methods

### Human samples

All study participants signed informed consent and the study was approved by University of California San Francisco (UCSF) and Ohio State University (OSU) Institutional Review Boards. Participants were eligible if they had available SCC and normal tissue available for study. To reduce the possibility that tumors from the same individual might be related clonally, we chose tumors from different anatomical locations when they were excised on the same day. Tumors excised on different dates also needed to be excised from different anatomical locations. Re-excisions were not included in the study. Tumor DNA was microdissected from formalin-fixed paraffin embedded tissue sections containing at least 70% tumor cells and the concentration was measured using TaqMan analysis [22]. Blood DNA was used as a source of normal reference DNA for loss of heterozygosity analyses.

### aCGH

We obtained aCGH profiles from a total of 305 tumors from 181 patients and these consisted of one actinic keratosis, 37 Bowen's disease, 45 KAs and 222 SCCs. We focused our subsequent analyses on 222 SCCs from 135 individuals. The cohort included 25 patients who had 3 or more independent tumors that were examined by aCGH (number of tumors, n = 104). Tumor genomic DNA (1000ng) and reference DNA (600ng) (Promega) was labeled with Cy3 and Cy5, respectively using random primers essentially as previously described [23], [24]. The labeled tumor and reference DNA was pooled and applied to Hum3.2 BAC arrays for 48 hours. The arrays contained 2464 BAC clones with an average resolution of 100 Mb. Analysis of the arrays was carried out using R/Bioconductor software [25], [26]. Prior to analysis the data was normalized with respect to GC content and geometrical position on the arrays [27]. Regions of equal copy number were defined by segmenting the data using circular binary segmentation (CBS) [28]. The scaled median absolute deviation (MAD) of the difference between the observed and segmented values was used to estimate the sample-specific experimental variation; samples with a MAD of greater than 0.2 (n = 18) were considered unsuitable for inclusion in the study. The gain and loss status for each probe was defined using the merged level procedure [29].

### Statistical analyses

For each autosomal arm, correlation coefficients based on log2 ratio values were computed for each pair of samples for those patients who had at least three independent SCC samples. Only those sample pairs were considered where at least one of the samples had 20% of clones with absolute value greater than 2 times sample MAD and another 20% below 2 times MAD. This ensured that the correlation was not driven by a flat sample profile. Only those arms were considered where there were at least 40 clones with non-missing values and at least 20 sample pairs in each of intra and inter groups; arms excluded from analyses include 2p, 3q, 4p, 5p, 6p, 6q, 7p, 7q, 9p, 9q, 10p, 10q, 11p, 12p, 12q, 15q, 16p, 16q, 17p, 18p, 18q, 19p, 19q, 20p, 21q and 22q. Two-sided, two sample t-tests were performed comparing the intra and inter patient groups for each of those arms where there was no significant difference in group variances. Brown-Forsythe version of the Levene-type test [30] was used to test for unequal group variances. The t-test p-values were then adjusted for multiple testing by Holm's method. Since a number of arms had unequal group variances, t-tests with Welch's approximation were also performed on each arm. Theoretical p-values were then adjusted by Holm's method. Genome-wide p-value was similarly computed by considering clones from all autosomes. There was no difference in group variances when considering whole genome. The correlation coefficients when considering individual arms and also whole genome had near normal distributions.

We identified clones having high within vs. between patient effects by estimating intraclass correlation coefficient (ICC) which captured the within-patient similarity. A random effects model *Y_ij_* = μ + α*_i _*+ ε*_ij_*, where the response variable is the CBS value with original log2ratio if a clone is an outlier in that segment, *j* and *i* represent the tumor and patient respectively, μ is an unobserved overall mean, α*_i_* is an unobserved random effect shared by all tumors in patient *i*, and ε*_ij_* is an unobserved noise term, was fit for each clone and ICC calculated as σ_α_^2^/(σ_α_^2^+σ_ε_^2^) where σ_α_^2^ and σ_ε_^2^ are the variances of α*_i_* and ε*_ij_* respectively.

### Microsatellite LOH analyses

Matched normal and tumor DNAs from 65 individuals were genotyped for 45 microsatellite markers mapping to regions of common chromosomal loss or gain. A total of 270 skin tumors were studied for microsatellite LOH analyses. For allelotyping, we chose microsatellite markers with a high degree of heterozygosity that can be readily quantified. To allow efficient amplification from fixed tissue we selected microsatellite markers with PCR product sizes less than 200 bp [12]. Fluorescently labeled, multiplexed PCR products were analyzed on an ABI 377 DNA sequencer using GeneMapper v3.7 (Applied Biosystems) in the OSU Comprehensive Cancer Center Nucleic Acids Shared Resource. An allelic imbalance ratio (R) in each tumor sample for each marker was calculated using a standard protocol: R = (TA/TB)/(NA/NB), where TA is a peak height from tumor DNA of the larger sized allele, TB is the peak height area from tumor DNA of the smaller sized allele, NA is the peak height area from normal DNA of the larger allele, and NB is the peak height area from normal DNA of the smaller allele. As described by others, when R was greater than 1.5 or less than 0.66, the sample was considered to have allelic imbalance [31], [32]. When R was between 1.25 and 0.85 the sample was considered to have no imbalance. Other values for R were treated as uncertain.

### Preferential imbalance analyses of microsatellite genotypes

Allelic imbalance data using microsatellite markers cannot simply be compiled across individuals, due to the heterozygosity of allele sizes across individuals each individual is likely to have a different combination of genotypes. We used a Bayesian/frequentist approach which we developed specifically for these data to determine if any given marker showed preferential allelic imbalance within and across patients (Text S1). In brief, we evaluated *patient-specific* odds of preferential imbalance as an indicator of randomness in a given individual using a Bayesian method. We then combined these odds into a sample to assess the evidence in favor of preferential imbalance in the general population using a frequentist method. A Wilcoxon rank sum test was then performed and all loci that rejected the null hypothesis at a 5% level of significance were deemed to show preferential imbalance.

### Allele-specific imbalance studies

We conducted quantitative genotyping of matched normal and SCC tumor DNA pairs using Sequenom MassARRAY Iplex gold genotyping technology. It is highly quantitative and is extremely sensitive for detection of allelic gains or losses in tumors and has been used for allelic imbalance studies [33]. All genotypes of poorer quality (aggressive calls) and those for whom a water sample had a strong call were eliminated from further analysis. Genotypes were also discarded from analysis if one of the two paired normal/tumor DNAs did not work resulting in genotypes included in analysis from 299 SCCs from 130 individuals for *rs13281615*, 110 SCCs for 70 individuals for *rs1447295*, and 175 SCCs from 84 individuals for *rs6983267*. Genotypes and peak area data for each allele were analyzed to identify regions of genomic imbalance between each matched normal and tumor DNA. An allelic imbalance ratio (R) to measure imbalance in each tumor sample for each SNP was calculated as described for the microsatellite LOH studies. Duplicate SNPs were included for quality control and two control samples and two no template controls were used. Chi-squared analyses were used to determine significance of observed allelic imbalances compared to expected 50∶50 imbalances indicative of random allelic imbalance.

## Supporting Information

Text S1 Supplementary methods.
(0.05 MB DOC)
