# Supplementary material for: Germline Variation Controls the Architecture of Somatic Alterations in Tumors
Source: PLoS Genet. 2010 Sep 23;6(9):e1001136. doi: 10.1371/journal.pgen.1001136 (PMC2944791; doi:10.1371/journal.pgen.1001136)
Supplement: Text S1 — Supplementary methods. (0.05 MB DOC) [file pgen.1001136.s001.doc]

**Text S1**

**Bayesian/frequentist approach for analysis of microsatellite data**

Let U represent the phenomenon that the allelic imbalances occur completely at random, particularly that type “A” and “B” imbalances are equally likely. Let *U^C^* denote the negation of *U*, that is, imbalances “A” and “B” are not equally likely which corresponds to the hypothesis of preferential imbalance. Consider now *N* eligible patients. For each patient *j*, *1≤j≤N*, we take *n_j_*, the number of tumors showing an imbalance, and *x_j_,* the number of tumors showing a type A imbalance at a given marker, where 0≤ *x_j_* ≤ *n_j_*. Consequently, there would be *n_j_-x_j_* type “B” imbalances. Let *D_j_ =* (*n_j_, x_j_)* denote the data collected from the *j^th^* patient. In terms of *U* and *U^c^*, we defined the patient-specific odds against preferential imbalance, denoted by *O_j_*, as:

$O_{j}=\frac{P(U|D_{j})}{P(U^{c}|D_{j})}=\frac{P(U|D_{j})}{1-P(U|D_{j})}$ (1)

where P(V|W) denotes the probability of the event “V” given the event “W”. The patient-specific odds, *O_j_*, varies from zero to infinity with values close to zero providing support for preferential imbalance (*U^C^*), large values favoring complete randomness (*U*), and values near one indicating indeterminacy, neither supporting *U* nor *U^C^*. In the logarithmic scale, large negative values of *ln O_j_* indicate preferential imbalance whereas large positive values favor complete randomness. Values close to zero provide no evidence in support of either U or U^C^. We adopted a Bayesian approach to determine *O_j_* at each marker.

In testing the hypothesis of preferential imbalance at a given marker across OTR individuals in a population, we performed a Wilcoxon rank sum test of the hypothesis

$H_{0}:\mu\geq\mu_{0}\text{ versus H:}\mu<\mu_{0}$ (2)

where μ is the average of the log-transformed odds in the OTR population and μ_0_ is a pre-set threshold. In this study, μ_0_ = 0. Consequently, markers for which the null hypothesis H_0_ is rejected at the 1% level of significance were deemed to be candidate loci for susceptibility variants.

To compute patient-specific odds we let the parameter θ represent the probability of a type “A” (or type “B”) imbalance, 0≤θ≤1. For any OTR individual, imbalances of type “A” or “B” are declared to occur completely at random if U holds:

$U:\theta-0.5|<\varepsilon=0.15$ (3)

Equivalently stated, we characterize a state of complete randomness (U) by letting 0.35<θ<0.65 and a state of preferential imbalance (U^C^) by θ≤0.35 or θ≥0.65.

In a given marker, we assume that *D_j_ =* (*n_j_, x_j_*) behaves as a binomial random variable. Precisely,

(4)

This implies that the likelihood function of θ given the data from patient *j* data *D_j_*, *L* (*θ; D_j_*) is

$L(\theta;D_{j})\alpha\theta^{xj}(1-\theta)^{nj-xj}x(1-\theta)^{nj-xj+b-1}$ (5)

For convenience, we assign a natural conjugate prior density for θ denoted by *h (θ*)*.* It follows that *h* (*θ)* is a beta distribution with parameters *a* and *b*, or *θ* ~ *beta* (*a, b*)*.* Furthermore, we assume that the departure from randomness is symmetric about 0.5 which implies that *a=b*. We determine *a* such that the prior probabilities P(U) ≈ P (U^C^) which yields a≈2.6. This reflects our prior belief that complete randomness is as equally plausible as preferential imbalance as a data-generating process.

From Bayes theorem, the posterior density function g (*θ |D_j_)* is derived as follows

$h(\theta|D_{j})\alpha L(\theta;D_{j})xh(\theta)\alpha\theta^{xj+a-1}x(1-\theta)^{nj-xj+b-1}$ (6)

showing that *h*(*θ|D_j_*) is a beta distribution with parameters *x_j_+a* and *n_j_-x_j_+b,* as expected. From *h*(*θ|D_j_*) we obtain the probability of randomness given the observed data, *P*(*U|D_j_*) by evaluating the integral:

$P(U|D_{j})=\int_{0.5-\varepsilon}^{0.5+\varepsilon} h(\theta|D_{j})d\theta=\int_{0.35}^{0.65} h(\theta|D_{j})d\theta$ (7)

We implemented a code in MATLAB to evaluate the integral in (7). The term *P* (*U|D_j_*) is the numerator in the expression of *O_j_* in (1). The denominator of *O_j_*, *P*(*U^C^|D_j_*) is obtained using the fact that *P*(*U^C^|D_j_*) *= 1- P*(*U|D_j_*).
